# Salinity and High Temperature Tolerance in Mungbean [*Vigna radiata* (L.) Wilczek] from a Physiological Perspective

**DOI:** 10.3389/fpls.2016.00957

**Published:** 2016-06-29

**Authors:** Bindumadhava HanumanthaRao, Ramakrishnan M. Nair, Harsh Nayyar

**Affiliations:** ^1^Plant Physiology, World Vegetable Center, South AsiaHyderabad, India; ^2^Vegetable Breeding – Legumes, World Vegetable Center, South AsiaHyderabad, India; ^3^Department of Botany, Panjab UniversityChandigarh, India

**Keywords:** salinity, high temperature, physiological mechanisms, Na and K uptake, cropping systems, mungbean

## Abstract

Biotic and abiotic constraints seriously affect the productivity of agriculture worldwide. The broadly recognized benefits of legumes in cropping systems—biological nitrogen fixation, improving soil fertility and broadening cereal-based agro-ecologies, are desirable now more than ever. Legume production is affected by hostile environments, especially soil salinity and high temperatures (HTs). Among legumes, mungbean has acceptable intrinsic tolerance mechanisms, but many agro-physiological characteristics of the *Vigna* species remain to be explored. Mungbean has a distinct advantage of being short-duration and can grow in wide range of soils and environments (as mono or relay legume). This review focuses on salinity and HT stresses on mungbean grown as a fallow crop (mungbean-rice-wheat to replace fallow-rice-wheat) and/or a relay crop in cereal cropping systems. Salinity tolerance comprises multifaceted responses at the molecular, physiological and plant canopy levels. In HTs, adaptation of physiological and biochemical processes gradually may lead to improvement of heat tolerance in plants. At the field level, managing or manipulating cultural practices can mitigate adverse effects of salinity and HT. Greater understanding of physiological and biochemical mechanisms regulating these two stresses will contribute to an evolving profile of the genes, proteins, and metabolites responsible for mungbean survival. We focus on abiotic stresses in legumes in general and mungbean in particular, and highlight gaps that need to be bridged through future mungbean research. Recent findings largely from physiological and biochemical fronts are examined, along with a few agronomic and farm-based management strategies to mitigate stress under field conditions.

## Introduction

Globally, agriculture productivity is inhibited by abiotic and biotic stresses, but abiotic stresses in particular (Gong et al., 2013) affect spreading of plant species across different environmental zones (Chaves et al., 2003). Under this situation, the widely accepted benefits of legumes in cropping systems are needed now more than ever (Arnoldi et al., 2014; Araujo et al., 2015). Legumes/pulses are very important food and feed crops, known for their health benefits (Arnoldi et al., 2014) vital ingredient of Indian and Mediterranean diets and considered staple in other regions (Vaz Patto et al., 2014), have high demand as forage for producing high-quality meat and milk (Boelt et al., 2014).

The changing climate is expected to worsen abiotic factors globally and adaptation strategies need to be established for target crops to specific environments (Beebe et al., 2011). Connect between different stress factors will likely surge harm to crop yields (Beebe, 2012). As observed, the average yield of temperate legumes has moderately improved in past half a century, with about a 45–50% increase for most legumes (Araujo et al., 2015). The highest yield increase (~100%) was realized in groundnut etc, which is still lower than the jump achieved by major cereals (~140%) (FAOSTAT, 2013). The emphasis in last decade was on exploring ‘cause-effect’ (bio-physiological and molecular, etc.) relationships of abiotic stress responses of a broad range of crop species (Araujo et al., 2015). Although physiology given insights on plant responses referring stress tolerance, further dissection on the genetic basis of tolerance traits through integration of system biologyage approaches is needed to dissect the traits and ultimately obtain larger benefits (Mir et al., 2012; Araujo et al., 2015).

A recent review (Araujo et al., 2015) highlights the latest research accomplishments in understanding abiotic stress responses in model food and forage legumes, and supports the development of legumes that are better adapted to environmental constraints. The review emphasizes the need to address current demands on modern agriculture and food production activities impaired by global climate change. It mostly focused on mainstream crops such as soybean, common bean, chickpea, pea, cowpea and forage legumes such as alfalfa, but apparently less on abiotic stresses tolerance of other grain legumes like mungbean and black gram, probably largely due to the sparse availability of information on the diverse stress responses and the mechanisms of tolerance in these legume species.

Mungbean [*Vigna radiata* (L.) R. Wilczek] is cultivated on >6 million ha in the warmer regions of the world and is one of the most important pulse crops. It is a short duration (65–90 days) grain legume having wide adaptability and low input requirements (Nair et al., 2012). Cultivation of the crop extends across wide range of latitudes (40° N or S) in regions with diurnal temperatures of growing season are > 20°C (Lawn and Ahn, 1985). India is the largest producer and consumer, and accounts for about 65 and 54% of the world acreage and production respectively. Like other legumes, mungbean fixes atmospheric nitrogen (58–109 kg/ha) in symbiosis with *Rhizobium*, which not only meet its own nitrogen need, but also benefits following crops (Ali and Gupta, 2012). It requires relatively less water than other legumes for good growth and is important for its high nutritional value and for improving soil fertility (Parida and Das, 2005). Mungbean contains very low levels of oligosaccharides (sugars influence flatulence), is a good protein source (~23%) with high digestibility and suitable as baby food (Ihsan et al., 2013).

Mungbean is one of the common legume in most tropical and subtropical regions and grown after harvesting wheat and before ensuing autumn crops, and has a major role in ensuring the nutrition security of developing countries such as India (Dhingra et al., 1991). Being rich in proteins, minerals and vitamins, it is an indispensable ingredient in majority of Indian diets. Regardless of such merits, mungbean often is cultivated on marginal soils with low inputs, that prone to numerous abiotic stresses that greatly hampers seed yield (Singh and Singh, 2011). Due to its short duration, in the Indo-Gangetic plains it easily fits into established cropping rotations of rice in *kharif* (monsoon) and wheat in *rabi* (winter), the major crops in the northern Indian states of Punjab, Haryana, and Uttar Pradesh.

Static mungbean yield in last decades is largely accounts for crop susceptibility to various biotic and abiotic stresses at different growth stages of the crop (Sehrawat et al., 2013a). Among them, salinity severely limits growth and yield worldwide; ~50 mM NaCl can cause >60% yield losses (Abd-Alla et al., 1998). It is expected that increased salinity will have an irresistible global effects, resulting ~50% loss of arable land by mid of the 21st century (Hasanuzzaman et al., 2012).

Thus there is a compulsion to continuously improve agricultural productivity of staple crops, legumes and specifically mungbean to ensure nutritional requirements for burgeoning human population, especially in developing countries. Mungbean often is grown on irrigated soils (as relay crop in cereal cropping systems) with salt deposits in upper layer due soil evaporation of water during dry season or from varying amounts of salinity in irrigation water. This accumulated salt decreases osmotic potential of soil, create water stress and imparts nutrient imbalances that trigger metabolic damage and cell death (Hasanuzzaman et al., 2013b).

In this paper, we focus on abiotic stress tolerance in legumes, particularly mungbean, and highlight any gaps that should be addressed in future mungbean research. We explore influence of salinity and HT on mungbean grown as a fallow crop (mungbean-rice-wheat) and/or as a relay crop in cereal cropping systems. Recent findings in physiology, biochemistry and molecular aspects of mungbean are presented along with several agronomic and farm-based mitigation strategies. We also briefly emphasize the role of exogenous protectants and growth-promoting microbes and the underlying physiological mechanisms for transduction of stress signals for salinity and HT stress tolerance.

Apart from salinity and heat stress, water deficit and waterlogging are also the key abiotic stresses that restrict growth, development and yield traits in mungbean, but they are outside the scope of this review. Consulting recent reviews/reports (Mirzaei et al., 2014; Araujo et al., 2015) for legumes and (Singh and Singh, 2011; Kumar et al., 2013; Naresh et al., 2013) for mungbean would provide necessary details.

## Salinity

Legumes are economically important crops and serve as sources of nutritious food, feed and raw-materials for humans, animals and industries respectively. Additionally, legumes have a symbiotic association with nitrogen-fixing rhizobia present in the root nodules, thus plants do not require external nitrogen sources. However, legumes are highly salt-sensitive crops, and a high concentration of Na^+^ and Cl^–^ ions around the root zone in water-scarce areas limits geographical range of legumes in arid and semiarid climates where evapotranspiration exceeds precipitation. Usually, salinity affects plants in two modes: osmotic stress and ion toxicity. However, for legume species particularly, there is a third mode: reduced nodulation by rhizobia, as salinity affects them either directly or indirectly. However, response of legumes/other plant species differ liable to prevailing conditions and extent of stress intensity. Therefore, it is necessary to enhance productivity of food grain legumes and to exploit valuable natural resources more efficiently to meet the demand for nutritious food from a growing population.

### Salinity and its Effect on Crop Plants

Salinity limits the output of food crops and growth reduction is the main morphological effect on many biochemical mechanisms of the plant. Plants under high saline unable take up adequate water for metabolic processes or maintain turgidity due to low osmotic potential. Naturally, salt-alkalinized soils are complex that include various ions creating soil-salt-alkalization complex (Läuchli and Lüttge, 2002). Alkaline salts (NaHCO_3_ and Na_2_CO_3_) were shown more damaging to plants than neutral salts (NaCl and Na_2_SO_4_) (Yang et al., 2007). Salt stress generally involves osmotic stress and ion injury (Ge and Li, 1990). Differential response of plants to salt and alkali stresses are largely due to high-pH associated stress (Munns, 2002; Li et al., 2012). Increased uptake of cations, such as Na, Mg, Ca, cause different kinds of nutritional imbalances leads to different ranges of toxicity. Under general NaCl toxic conditions, plants absorb a higher amount of Na, which thus decreases the K Na^–1^ ratio (Ahmad and Umar, 2011; Ahmad and Prasad, 2012). In this review we focus and discuss only NaCl-induced salinity effects on general plant response.

#### Salinity Effect: Physiological and Biochemical Mechanisms

Salinity stress involves changes in various physiological and metabolic processes, varies with stress severity and its duration and ultimately inhibits crop production (Munns, 2005; Rozema and Flowers, 2008). Initially, soil salinity represses plant growth through osmotic stress, which is then followed by ion toxicity (Rahnama et al., 2010; James et al., 2011). During initial phases, the water absorption capacity of the root system decreases and water loss from leaves is accelerated due to osmotic stress, and therefore salinity stress is also considered hyperosmotic stress (Munns, 2005). Osmotic stress at the initial stage causes various physiological changes, such as interruption of membranes, nutrient imbalance, impaired ability to detoxify reactive oxygen species (ROS), differences in antioxidant enzymes, and decreased photosynthetic activity (Munns and Tester, 2008; Pang et al., 2010). One of the most damaging effects is accumulation of Na^+^ and Cl^–^ ions in tissues of plants exposed to soils with high NaCl concentrations. Higher Na^+^ blocks K^+^ uptake, results in lower productivity and may even lead to cell death (Ahmad and Umar, 2011; James et al., 2011).

**FIGURE 1:**
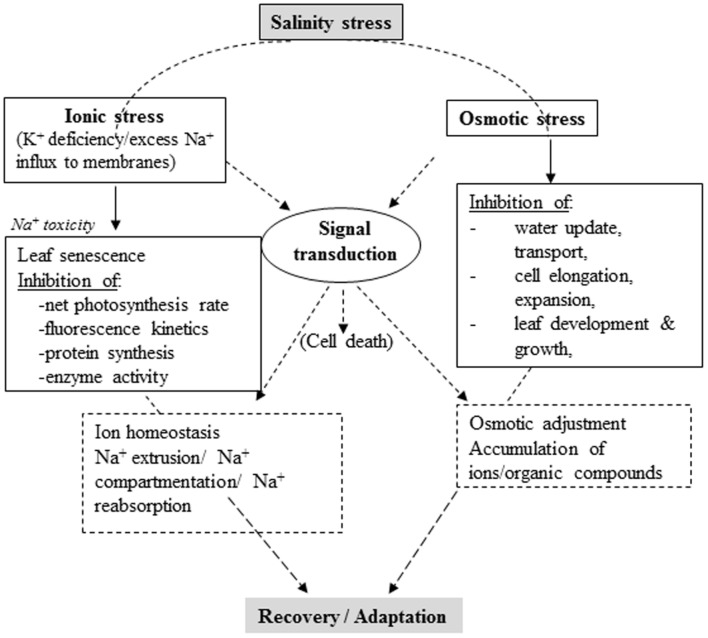
**Schematic summary of salinity stress in plants and corresponding intrinsic physiological responses (Partly adapted from Horie et al., 2012)**.

Plant adaptation or tolerance to salinity stress involves complex physiological traits, metabolic pathways and molecular or gene networks. Comprehensive knowledge of how plants respond to salinity stress at different levels and an integrated approach combining molecular, physiological, and biochemical techniques (Palao et al., 2014) are imperative for developing salt-tolerant varieties in salt-affected areas (Ashraf, 2014). Recent research revealed various adaptive responses to salinity stress at cellular, metabolic and physiological levels, although mechanisms underlying salinity tolerance are yet to be clearly understood (Gupta and Huang, 2014). Plants develop various physiological and biochemical mechanisms to survive in soils with high salt concentration. Principal mechanisms include, but are not limited to, ion transport, uptake and compartmentalization biosynthesis of osmoprotectants and compatible solutes, activation and synthesis of antioxidant enzymes/compounds, polyamines and hormonal modulation (Reddy et al., 1992; Roy et al., 2014) (**Figure 1**).

#### Salinity and Hormonal Regulation

Among the plant hormones, ABA plays a key role with ameliorating stress effects. As a first line of defense, ABA has long been recognized to synthesize in roots during soil water deficit (Popova et al., 1995; He and Cramer, 1996). ABA can mitigate inhibitory effects of salinity on photosynthesis (Yong et al., 2014), growth, and translocation of assimilates (Cabot et al., 2009). The positive link of ABA with salinity tolerance partially credited to K^+^, Ca^2+^ levels (Hussain et al., 2016) and compatible solutes in cytocol, which offset Na^+^ and Cl^–^ uptake (Chen et al., 2001; Gurmani et al., 2011). Other compounds such as, salicylic acid (SA) and brassinosteroids (BR) also share abiotic stress responses (Fragnire et al., 2011; Khan et al., 2015). Ashraf et al. (2010) reviewed a possible role of BRs and SA in mitigating harmful effects of salt stress and discussed their exogenous applications in regulating various biochemical and physiological processes. Reddy et al. (2015) suggest over-production of proline eases salt stress and protects photosynthetic and antioxidant enzyme activities in transgenic sorghum [*Sorghum bicolor* (L.)]. The effect of salt stress on intrinsic physiological traits, malondialdehyde (MDA) levels and antioxidant enzyme activities were evaluated in 40-day-old transgenic lines and compared with wild type plants. The photosynthetic rate was reduced in wild type plants almost completely. Salinity induced cent percent stomatal closure in wild type, while it did only 64–81% in transgenic plants (after 4 days), indicating transgenic lines were better in coping up with salt stress than wild type.

### Salinity: Effect on Physiological Traits

Largely in response to salt stress, crop varieties/genotypes vary in their inherent ability to adjust several physiological and biochemical processes (Grewal, 2010; Singh et al., 2014). Most observed both physiological (high Na^+^ transport to shoot, favored storage of Na in older leaves, high Cl^–^ uptake, lower K^+^ uptake, low P and Zn uptake, etc.) and biochemical (alteration in scavenging enzymes synthesis and their expression, rise in non-toxic compatible solutes, etc.) and their modifications. Ashraf (2014) described agronomic characters that represent, combined effects (G × E) on plant growth, includes growth rates and physiological efficiencies related to salinity tolerance. Physiological traits lead to more reliable evidence than agronomic traits; thus, several reports mainly focused on water relations, carbon assimilatory functions and synthesis of various inorganic compounds and organic solutes in discrete crop species.

For assessing response to various abiotic stresses, effective indicator is yield; however, it may not capture underlying genetic mechanisms linked to strong environmental interaction. Intricate inheritance pattern and low heritability limit selection efficacy for yield or biomass under abiotic stresses (Ashraf, 1994; Noreen et al., 2010). Furthermore, dearth of a dependable trait for screening hinders developing salt tolerance in target crops (Palao et al., 2014) which is also true in mungbean (Mahdavi and Sanavy, 2007). Instead, it would be more sensible, if one can focus of using simply inherited morpho-physiological traits inked to yield. However, using physiological traits as selective markers to achieve success rest on strength of the relationship of such markers with salinity response of target plants (Roy et al., 2014). Yet, selection from pool of markers would be more effective than rely on a single marker. Tolerance of a genotype was found linked to its ability to confine assimilation potential toxic ions (such as Na^+^) and better balancing of ions (such as K^+^). Adding K^+^ to nutrient solution reduced negative effect of NaCl on growth in barley (Roy et al., 2014). Although uptake of both Na and K are distinct, lower Na/K ratio is treated desirable. Na^+^ is transported to shoots from roots typically through passive uptake, while K^+^ through the active uptake (Hasegawa et al., 2000).

At entry level, plant roots experience salt stress when Na^+^ and Cl^–^ along with other cations are present in the soil in varying levels. Ion uptake depends upon the plant growth stage, genotype, temperature, RH and light intensity (Singh et al., 2002). Salt in excessive amount retards plant growth, decrease yield and may cause plant death (Garciadeblás et al., 2003). It also induces production of ROS/intermediate viz., superoxide, H_2_O_2_ and OH^–^-radicals in chloroplasts and mitochondria. Plants have devised different systems for scavenging ROS by using enzymes such as SOD peroxidases and catalases. Under normal growth condition, ROS in cells is as low as 240 μMs^–1^, however, under salinity, ROS production reaches to 720 μMs^–1^ (threefold) (Mittler, 2002; Mudgal et al., 2010). H_2_O_2_ concentration of 10 μM reduced net photosynthesis rate by 50%. Identifying a single criterion as an effective selection target is difficult, given the complexity of salt tolerance. However, some information is available on the use of these attributes as selection criteria for improving salt tolerance through breeding.

### Salinity and Mungbean

Salinity stress causes a significant reduction in mungbean yield (Abd-Alla et al., 1998; Saha et al., 2010) through decline in seed germination, root and shoot lengths, fresh mass and seedling vigor and varies with different genotypes (Promila and Kumar, 2000; Misra and Dwivedi, 2004). Salt injury also leads to pronounced symptoms like enhanced chlorosis, necrosis and decreased content of chlorophyll and carotenoids in mungbean (Gulati and Jaiwal, 1993; Wahid and Ejaz, 2004). NaCl stress had more deleterious effect on roots than shoots, with a sudden dip in root growth associated traits (Friedman et al., 2006; Saha et al., 2010).

Most of the mungbean cultivars tolerate salt to an extent of 9–18 m mhos/cm than usual salt concentration of 5–6 m mhos/cm (at germination stage). Paliwal and Maliwal (1980) reported that mungbean seeds could tolerate 6 m mhos/cm salinity, compared to 3 m mhos/cm for black gram. In another study, 42 cultivars of mungbean and black gram were tested at five levels of salinity (3–18 mmhos/cm) in 1/5 Hoagland nutrient solution in plastic containers (Maliwal and Paliwal, 1982). Germination/seedling length of all the cultivars was delayed/decreased; salinity and its tolerance limit varied with the cultivar. Some varieties (S 72, H 45, No. 525, Madira and RS-4) were found to be more salt-tolerant. Mungbean accumulates compatible solutes like proline and betaine to mitigate damaging effects (Hoque et al., 2008), however, not at a high level to regulate osmotic potential in plants (Hamilton and Heckathorn, 2001; Hossain and Fujita, 2010). In one of the study (Phillips and Collins, 1979), callus from mungbean grown in sand culture with Hoagland’s nutrient solution supplemented with 0–350 mol/m^3^ NaCl, showed tolerance to salt as that of whole plant, suggesting mungbean appears to have salt tolerance at cellular level.

In a field experiment, Reddy (1982) observed injury to mungbean cultivars with irrigation water containing EC of 4 m mhos/cm. More and Ghonikar (1984) determined that the critical level of salinity in irrigation water to cause injury to seed germination in mungbean was 3.5 m mhos/cm. We also observed similar results while field screening 45 elite mungbean accessions with different sources of irrigation water under field conditions in Punjab, India. The irrigation water (tube well water with 2200 μmhos EC and residual sodium carbonate – RSC of 6.4 meqL) resulted in growth retardation at the vegetative stage compared to irrigation (canal) water with EC of 290 μmhos and RSC of 0.7 meqL^–1^ (Bindumadhava et al., 2015). Lawn et al. (1988) reported from a pot study that some of the accessions of *V. radiata* var. *sublobata* showed no symptoms of chlorosis when grown on extremely alkaline (pH > 8.5) calcareous soils. Roychoudhury and Ghosh (2013) studied the physiological and biochemical responses of *V. radiata* seedlings to varying concentrations of cadmium chloride (CdCl_2_) and NaCl. Both chemicals enhanced seedling mortality, notably at higher concentrations. A decline in normal growth, germination %, inhibition in root and shoot length and decreased fresh and dry weights of seedlings was observed. The activities of antioxidant enzymes, catechol-peroxidase and catalase increased progressively with an increase in CdCl_2_ and NaCl concentrations. Altering the level of biomolecules and modulating physiological and biochemical functions, *V. radiata* seedlings could overcome the cellular toxicity of CdCl_2_ and NaCl (Roychoudhury and Ghosh, 2013). Degree of salt stress affects different crops differently. For mungbean, RSC beyond ~ 4 meq/L is considered moderate salinity while more than 7 meq/L is very high. Similarly, a pH of 8.5 – 9.1 is considered as non-stress, 9.2 – 9.8 as moderate stress, and ≥9.8 as high stress. Extremely high salt stress conditions damage the plant, but moderate to low salt stress affects plant growth rate and thereby manifests symptoms that could be associated with morphological, physiological, or biochemical change (Hasegawa, 2013). Singh et al. (1989) reported that four mungbean cultivars in plots salinized with 2,4, and 6 dS m^–1^ gave average seed yield of 906, 504, and 370 kg/ha, respectively. Salinity occupies a prominent place among the soil problems that threaten the sustainability of agriculture in Pakistan. Out of 16.2 m ha of land under irrigation, more than 40,000 ha of land are lost each year (Yasin et al., 1998). Mungbean is planted in annual crop rotations on an increasingly large area of heavy clay soils in many regions of Pakistan frequently exposed to moderate to high levels of salts. Mungbean production (455 kg/ha) in Pakistan is very low compared to other countries (Agricultural Statistics of Pakistan, 2001). The major mungbean-growing areas in the country are affected by salt and to make effective use of salt-affected soils, it is important to select mungbean genotypes that can tolerate salt stress and produce substantial yields under saline environments.

#### Salinity Effect at Different Growth Stages of Mungbean

##### Germination and seedling growth

During germination under saline conditions, high osmotic pressure of saline water is created due to capillary rise leading to more salts density at seed depth than at lower soil profile, which reduces time and rate of germination (Mudgal et al., 2010). In mungbean seedlings, high salt concentration causes increased H_2_O_2_ content in both roots and leaves, hence salts should be removed to ensure proper growth and development (Saha et al., 2010). Both root and shoot lengths were reduced with increased NaCl concentration, but roots were more damaged, with an increase in number of lateral roots and increase in its thickness, compared to shoots (Misra et al., 1996). Photosynthetic activity of mungbean is reduced due to reduced function of electron transport and instability of pigment protein complex (Promila and Kumar, 2000). High salinity results in a decrease in total leaf area and stomatal opening (Nandini and Subhendu, 2002). Proline and glycinebetaine levels in roots and shoots increased in mungbean (tolerant) cultivar ‘T 44’ subjected to NaCl stress at seedling stage (Misra and Gupta, 2006). Increase was seen with a supply of 5 mM CaCl_2_ to 200 mM NaCl. Calcium ions play a key role in osmoprotection and effects of Na^+^ and Ca^2+^ are thus harmonizing the accretion of osmolytes (Hu and Schmidhalter, 2005). Increased proline levels occurred when proline oxidase activity was low and high production of P-5-CR and y-glutamyl kinase in both roots and shoots. Thus, calcium facilitated osmolytes synthesis in NaCl-stressed mungbean seedlings (Misra and Gupta, 2006). When three species of *Vigna (V. radiata, V. mungo*, and *V. unguiculata)* subjected to varied doses of NaCl (50, 75, 100, 125, and 150 mM), reduction in chlorophyll content, sugar, starchand peroxidase enzyme activity were observed in shoots and roots (Arulbalachandran et al., 2009). Germination %, seedling growth rate, RWC and photosynthesis decreased with increasing NaCl levels in all species. The growth decrease was higher in mungbean than in black gram and cowpea. However, increase of compatible solutes was higher in cowpea than in black gram and mungbean, suggesting cowpea is more salt-tolerant than other two. The effect of pre-soaking seed in 50, 100, and 1000 μM SA on growth parameters of two mungbean genotypes (NM 19-19 and NM 20-21) under salinity stress (50 and 100 mM NaCl) was studied (Shakeel and Mansoor, 2012) and found a reduced seedling length and fresh/dry weight of both genotypes. Pre-soaking treatments (100 μM) with SA reduced salinity-induced decline. However, pre-treatment with a high concentration (1000 μM) prior to salt treatment caused a significant reduction in mean seedling length. NM 19-19 respond better under salt stress than NM 20-21. In a pot experiment (from Bangladesh), effect of salinity levels (e.g., 0, 0.1, 0.2, 0.3, and 0.4% of NaCl) on germination, growth and nodulation of mungbean varieties (BARI Mung 4, BARI Mung 5 and BARI Mung 6) was observed. Salinity affected germination and root elongation. Root growth was significantly reduced with higher salt and BARI Mung 4 showed better performance than other varieties. All showed similar performance in yield traits at higher NaCl levels. No effect on nodulation at a higher (0.4% NaCl) dose was seen in BARI Mung 5. However (Naher and Alam, 2010), reported nodules per plant decreased but not nodule size with increase in salinity.

##### Nodulation and nitrogen metabolism

Salinity interfered with initiation, reduced number, weight and nitrogen-fixing efficiency of nodules in chickpea, cowpea and mungbean, (Balasubramanian and Sinha, 1976), causing significant reduction in leghaemoglobin content, which decreased with aging of nodules by irreversible oxidation. Inhibition of root colonization by *Rhizobium* was the main reason for poor nodulation (Mudgal et al., 2010). Although nodules were present, nitrogen fixation was inhibited completely in inoculated plants grown at 6 dS m^–1^. These findings largely indicate process of symbiosis is salt-sensitive than both *Rhizobium* and the host plant (Zurayk, 1998). Plants in saline habitat accumulate proline and glutamine and increase concentration of amino di-carboxylic acid (Soussi et al., 1998; Munns et al., 2002). The ill effect on nitrogen metabolism is mainly observed in above ground plant parts (Munns et al., 2006; Munns and Tester, 2008). Symbiotic association of rhizobia with legume roots has specific advantages during different abiotic stresses; rhizobia aid N_2_ fixation and facilitate water uptake, thereby helping withstand heat stress. Some strains of rhizobia which are resistant to salts, helped in solubilizing osmolytes/ions in the rhizosphere and thus improves salt tolerance. *Rhizobium* and *Bradyrhizobium* spp. vary in their tolerance to salinity, though they nodulate the same plant differ in salinity tolerance (Elsheikh and Wood, 1995; Elsheikh, 1998) The effect of salinity on growth and survival of *Rhizobium* spp. suggests growth of these tested strains and species decreased when EC was raised from 1.2 to 6.7 mS cm^–1^ or to 13.1 mS cm^–1^ (Kaur D. et al., 2015), indicating that many strains of *rhizobia* can grow and survive at salt concentrations that inhibitory to most agricultural legumes. Hence focus should be more on exploiting salinity effect on symbiosis rather than survival of *Rhizobium* spp.

##### Growth, flowering yield pattern

Soil salinity delays and also reduces flowering and yield of crop plants (Maas, 1986). Salinity-induced reduction in yield was reported in many crops viz., wheat, barley, mungbean and cotton (Keating and Fisher, 1985). Mungbean showed decreased growth, photosynthesis and yield at a high salinity, but postponed pod ripening during the spring resulted in reduced pod shattering (Sehrawat et al., 2013a,d). A study by Hasanuzzaman et al. (2012) on screening mungbean germplasm for salt tolerance in the spring season identified a few resistant genotypes for saline areas. NaCl stress, combined with other types of stress, resulted in organ-specific changes in polyamine content in mungbean plants and affected enzyme activity. An excessive amount of salt can enter transpiring stream and cause injury to leaves, resulting in reduced photosynthesis of growing leaves (Mittler, 2002; Okuma et al., 2002; Tilman et al., 2002; Neelam and Dwivedi, 2004; Hossain and Fujita, 2010). Misra and Dwivedi (1995) reported a salt-tolerant cultivar was characterized by higher levels of total soluble carbohydrates than a salt-sensitive cultivar irrespective of salinity level. The physiological processes were compared in salt-tolerant (‘Pusa Vishal’) and sensitive (‘T 44’) mungbean cultivars and how SA effective in alleviate reduction in photosynthesis under salt stress (Nazar et al., 2011) was also explored. SA (0.5 mM) found effective as osmoprotectant as it increased nitrogen and sulfur assimilation and restricted Na^+^ and Cl^–^ content in leaves, maintained higher photosynthetic efficiency in ‘Pusa Vishal.’ However, diverse accessions resistant to salt stress within *Vigna* species could be useful source for exploring mechanism of salt tolerance was proposed (Zahir et al., 2010; Win et al., 2011). Saha et al. (2010) reported that pre-treatment with a sub-lethal dose of NaCl was able to overcome adverse effects of stress imposed by NaCl. Mungbean plants could acclimatize to lethal levels of salinity through pre-treatment with sub-lethal doses, which resulted in increased growth and photosynthesis in seedlings and modified the activities of antioxidant enzymes. Bourgault et al. (2010) demonstrated mungbean as a better adapted species to semi-arid and arid climates and able to maintain its photosynthetic rates, harvest index under water deficit and NaCl stress, than common bean. Further, addition of K^+^ was also examined to alleviate the stress effect and found higher levels of K^+^ improves water retention, growth and yield of mungbean (Wahid and Ejaz, 2004). In a separate study, Sehrawat et al. (2015) reported the effect of salt stress (two levels: 50 and 75 mM NaCl) on two mungbean varieties, ‘Pusa Vishal’ and ‘Pusa Ratna,’ during summer season and found significant variations and adaptability in both varieties. The plants in early growth stages were more resistant compared at reproductive stages (Sehrawat et al., 2013b,c). Salinity and associated osmotic stress severely constrained plant growth, physiology and yield traits. In general, the tolerant cultivar ‘Pusa Vishal’ exhibited less reduction in growth and yield traits than ‘Pusa Ratna.’

Globally, ongoing research to address salt-related problems are based on: (a) changing the growing environment suitable for normal growth of plants, or (b) selecting crop and/or altering genetic makeup, so that it can be grown in such saline areas. The first method involves major tweaking in systems biology and soil amelioration, which demand considerable resources and are often out of reach. The second approach (biological; developing crop varieties tolerance to salts) is more promising, economical, and socially acceptable^1^. Plants with high salt tolerance allow farmers to optimally manage their available resources. The latter approach is being attempted in mungbean by identifying intrinsic salt-associated traits and putative salt-tolerant lines from among world vegetable center’s core and mini-core mungbean collections (Schafleitner et al., 2015) and further, developing salt-tolerant accessions to use as donor sources in future breeding programs (Bindumadhava et al., 2015). Once developed, such selections can be validated in saline soils in the field for survival, growth and yield performance. A third “fusion” approach combines modified environmental and biological methods, which is highly prolific, less resource intense and frugally viable. Current global soil reclamation programs involve both biology and fusion approaches to contest salinity-associated problems.

Salt-tolerant wild mungbean (*Vigna marina*, beach cowpea) is found on tropical and subtropical beaches around the world (Hawaii and islands in the Pacific Ocean, the Caribbean, along the Atlantic and Indian Ocean coasts of Africa, India and Sri Lanka, Australian coasts^2^, United States Department of Agriculture [USDA] (2011). It has been domesticated, adapted and released as a dual-purpose legume in Bangladesh. *V. marina* is considered a light-sensitive type with profused vegetative growth habit showing tolerance to saline conditions (Hamid and Haque, 2007). Chankaew et al. (2014) reported quantitative trait loci (QTLs) for mapping salt tolerance in *V. marina*. QTL mapping in the F_2_ population (*V. luteola × V. marina* subsp. *oblonga*) revealed that salt tolerance in *V. marina* subsp. *oblonga* is controlled by a single major QTL, however, segregation analysis indicated it is controlled by a few genes. *Vigna marina* would be a potential salt-tolerant donor for future *V. radiata* breeding programs (inter-species hybridization can be attempted) for its natural growing habitat and intrinsic salt resistance mechanisms.

### Salinity Tolerance in Legumes from Molecular Perspective

In a review on salt tolerance in Asiatic *Vigna* species, Mishra et al. (2014) opined transgenic approach had made limited progress due to severe recalcitrance of these plants to genetic transformation and *in vitro* regeneration. Strategies discussed were overexpression of genes involved in (a) exclusion of toxic sodium ions by plasma membrane Na^+^/H^+^ antiporter; *SOS1* in legumes (Kido et al., 2013), (b) sequestration of sodium ions in vacuoles to reduce ionic toxicity by isolation and characterization of vacuolar Na^+^/H^+^ antiporters of legumes (Li et al., 2006), (c) encoding for compatible osmolytes by raising osmotic potential of cells (Porcel et al., 2004), (d) encoding for antioxidant enzymes to oxidative stress (Jiang et al., 2013). Role of regulatory genes include (a) upregulation of calcium in response to stress; isolation and characterization of novel calcineurin B-like proteins interacting with protein kinases (CIPKs) (Imamura et al., 2008) and (b) manipulation of transcription factors including AP2/ERF, bZIF, NAC, MYB, MYC, Cys_2_His_2_ zinc-finger and WRKY (Bhatnagar-Mathur et al., 2008). Use of activation tagging in model legume, *Medicago truncatula* for gene discovery, could extend to identify novel genes in Asiatic grain legumes (Scholte et al., 2002). RNAi mediated inactivation of a transcription factor of *M. truncatula*, MtNAC969, known to be associated with salt stress (de Zélicourt et al., 2012) is another strategy. However, gene-expression with constitutive promoters provide partial biological information than inducible or cell type-specific promoters. Choice of promoters can significantly affect the output from transgenic manipulation. Further, several findings on transformation for improving salinity tolerance focus toward genes controlling ion transport, as regulation of Na^+^ uptake and compartmentalization is critically important for plant survival and many candidate genes controlling this mechanism have been identified (Gupta and Huang, 2014).

Genetic variability and differential responses to stress enable to identify physiological mechanisms, sets of gene/s products that would impart stress tolerance and facilitate in incorporating under suitable background species to develop salt tolerant-types.

### Management Practices for Alleviating Salinity under Field Conditions

In the field, if the salinity level is less intense, the crop show lopsided in plant vigor. Moderate salinity, if uniform, display the restricted vegetable and reproductive growth without apparent injury *per se*. Leaves in salt-infested areas may appear smaller and darker blue-green in color (Richards, 1954).

Through avoiding leaching of salts from root zones, systematic monitoring farm management activities, and planting saline tolerant crop species, salinization can be controlled. Irrigation in agriculture can be sustained by efficient water management techniques (partial root zone drying methods, and by drip irrigation, etc.). Dry land salinity can be mitigated by reducing water infiltration beyond the root zones, which may restore the balance between water fall and water use, thus averting rising water tables and movement of salt to soil surface (Manchanda and Garg, 2008). Farming systems can alter in incorporating perennials in rotation with annual crops (phase-wise and rotation farming), in mixed plantings (inter-cropping), or in site-specific planting (precision farming) (Munns et al., 2002). Evolving efficient, low cost, easily adaptable methods for abiotic stress management is a major challenge. Worldwide, extensive studies are being conducted to develop strategies to cope with abiotic stresses through the development of salt- and drought-tolerant varieties, by shifting crop calendars, and by improving resource management practices (Venkateswarlu and Shanker, 2009)^3^. The following approaches would aid in removing/reducing salts:

#### Soil Reclamation

Salt removal from root zone is perhaps most effective and long-lasting way to ameliorate or even eliminate detrimental effects of salinity. It involves substituting sodium in the soil with calcium ions (through applying large quantities of gypsum). The released sodium ions are then leached deep beyond the root zone using excess water and finally moved out of the field through drainage. Gypsum slowly mix with water releasing calcium ions, which replace sodium ions from the soil into the downward moving water (Ramajulu and Sudhakar, 2001)^4^.

#### Suitable Use of Ridges or Beds for Planting

Impact of salinity may be minimized by sowing seed or planting seedlings on ridges effectively. Planting plants on ridge shoulder than ridge top is effective in escaping from salt episodes as during water evaporation, salts concentrate only at top rather on shoulders thereby minimizing salt ill effects. However, for alternate furrow planting, ideal would be planting on one shoulder of the ridge closer to water source. (Goyal et al., 1999a,b)^4^.

In a recent review, Shrivastava and Kumar (2015) explained how a range of adaptations and mitigation strategies help to cope with global salinity impacts^3^. These plans assume cost- and time-intensive but assures strength in developing simple, low-cost natural means for handling salinity episodes better in future soil management strategies.

#### Broad Management Practices to Reduce Salinity Impact

Additionally, following approaches may help in reducing negative effects of salinity (Qureshi et al., 2003).

(a) Adding crop residues/green manure as organic mulch improves soil health, structure, and water penetration, thus protects from adverse salinity effects. Steady mixing of organic matter to soil (crop residue, sludge and compost etc.) would reduce soil evaporation which lessens upward salt movement. Low evaporation demands less water which helps in lower accumulation of salts.

(b) Salt accumulation closer to the surface is a typical feature of saline soils. Deep tillage would mix salts present in surface zone into a much larger soil volume and reduce salt content and impact. Many soils have an impermeable hard pan, which hinders salt-leaching process. Under such circumstances, “chiseling” would improve water infiltration and the downward movement of salts^4^.

## High Temperature

### High Temperature and its Effect in Crop Plants

The global air temperature is predicted to rise by 0.2°C per decade, which will lead to temperatures 1.8–4.0°C higher than the current level by 2100 (Intergovernmental Panel on Climate Change [IPCC], 2007)^5^. HTs are injurious to plants at all stages of development, resulting in severe loss of productivity (Lobell and Asner, 2003; Lobell and Field, 2007; Hasanuzzaman et al., 2010). In response to unfavorable temperatures, plant biomolecules (stress proteins, enzymatic and non-enzymatic antioxidants, osmolytes and phytohormones) come into rescue (Kaur R. et al., 2015). Typically, their endogenous levels shoot up as plant defense and expression depends on type of plant species exposed, and intensity/duration of the stress. Legumes show varying degrees of sensitivity, which reduces their potential performance at different developmental stages such as germination, seedling emergence, vegetative phase, flowering and pod/seed filling phase (Bhandari et al., 2016). To address ever-fluctuating temperature extremes, efforts are being made in developing tolerant genotypes in legume either by conventional breeding strategies and/or more newly, by molecular breeding methods.

#### High Temperature Effect on Photosynthesis and Growth

High temperatures can constrain growth, lower yield and truncate crop cycles (McKenzie et al., 1988; Araujo et al., 2015). Temperature range which is not sufficiently high to damage cells, may inhibit growth associated tissue water functions and carbon assimilation associated chloroplast functions coupled with impaired vigor, cellular respiration, N fixation and metabolism (Buxton, 1996).

Among several processes, photosynthesis (Ps) is one such important physiological process impaired by heat stress (Crafts-Brandner and Salvucci, 2002). HT impacted more on photosynthetic capacity of C_3_ plants than C_4_ (Yang et al., 2006). In chloroplasts, carbon metabolism and photochemical reactions are considered primary sites of injury (Wang et al., 2009). Major effect such as altered structural organization of leaf stromal and thylakoids assembly and functions under heat stress (Rodriguez et al., 2005).

The maintenance of leaf gas exchange (transpiration and CO_2_ assimilation rates) under heat stress has a direct relationship with heat tolerance mechanisms of active leaves (Kumar et al., 2005). Heat markedly affects the leaf stomatal and CO_2_ assimilatory functions driven by photosystem II-photochemistry (Greer and Weedon, 2012). The mean change in photosynthesis capacity of *Vitis vinifera* leaves was declined by 60% with growing temperature from 25 to 45°C. Heat imposes negative impacts on leaves, such as reduced water potential, leaf area and pre-mature senescence and drop in photosynthesis (Greer and Weedon, 2012). Other likely reasons believed to hamper photosynthesis under heat stress are reduction in soluble proteins, RuBisCO binding proteins, large-subunits (LS), and small-subunits (SS) of RuBisCO in darkness, and increases of those in light (Sumesh et al., 2008)^3^. HT also affects starch and sucrose synthesis through reduced activity of sucrose phosphate synthase and ADP-glucose pyrophosphorylase (Rodriguez et al., 2005). Loss of productivity in heat stress is chiefly related to decreased assimilatory capacity by altered membrane stability (Zhang et al., 2006), enhanced maintenance respiration (Reynolds et al., 2007), and reduction in radiation use efficiency.

Among different growth stages, firstly germination is affected followed by other associated process related to seed hormones, though it is species specific and temperature range dependent (Johkan et al., 2011). Reduced germination percent, seedling emergence, cell size, poor vigor, reduced radicle and plumule growth were major impacts of heat stress reported in various plant species (Rodriguez et al., 2005; Piramila et al., 2012). Since there is a commonality in stress response at the cellular level, it is likely that thermo-tolerant lines might show tolerance to other stresses such as desiccation and salinity (Kumar et al., 1999). In common bean morphology (plant growth habit and height, leaf shape, – and physiological traits (phenology, water relations and shoot growth) were hampered by heat stress (Koini et al., 2009). Under excess heat stress, plants exhibit programmed death of specific cell and tissue types. Conversely, moderate HTs for extended duration leads to gradual death. Both types of injuries can lead to leaf shedding, flower/pod drop, abortion of reproductive organs and premature maturity or (Hasanuzzaman et al., 2010). HT also results in low yield, plant quality and seed viability (Anderson and Sonali, 2004; Khalil et al., 2009). In chickpea, it was shown that heat stress induced reproductive failure that would possibly impair sucrose metabolism in leaves, anthers and inhibit sucrose transporters; consequently, availability of trios phosphates (reducing and non-reducing sugars) to developing pollen would be upset, impairing its functions and causing reproductive failure (Kaushal et al., 2013). In model crops, such as *Arabidopsis* and tomato, tangible heat sensitive growth stage in crop cycle was elucidated and relevant proteins involved and their functions were described (Scharf et al., 2012). However, its implication for other legumes needs to be revealed to exploit trait-linked functionality. More recently, Gaur et al. (2015) highlighted that heat stress during reproductive stages is becoming a serious constraint to grain legumes productivity as their cultivation is expanding to warmer environments and temperatures are increasing due to climate change.

Plants characteristically have number of adaptive, avoidance or acclimation mechanisms to face HT. However, their survival depends on ability to perceive HT stimulus, generate and transmit the signal and initiate appropriate cellular, physiological and biochemical changes. Interestingly, breeding strategies aimed at enhancing drought tolerance will often capture plant responses to heat stress; hence heat tolerance may not be a specific target for improvement by most plant breeders (Hasanuzzaman et al., 2013a). Plants continuously tussle for survival under HT and alter their metabolism in various ways, particularly through compatible solutes and modify antioxidant systems (Munns and Tester, 2008).

#### Heat Stress Tolerance from Cell Signaling and Molecular Perspectives

As a multi-genic trait, several mechanisms are involved in heat tolerance, includes antioxidant activity, membrane lipid unsaturation, gene expression and translation, protein stability and accrual of compatible solutes (Kaya et al., 2001). In a review on heat stress, Bita and Gerats (2013), discussed activation of lipid based signaling cascades, increased Ca^2+^ influx (Saidi et al., 2011), heat shock proteins (HSPs) acting as molecular chaperones to prevent denaturation/aggregation of target proteins and facilitating protein refolding (Lohar and Peat, 1998) and transcriptional repression by repetitive DNA regions (Khraiwesh et al., 2012). Heat shock factors (HSFs) are transcriptional activators of HSPs (Banti et al., 2010). For e.g., in *Arabidopsis*, expression of APX gene family is heat stress dependent and regulated by HSF that links heat stress response with oxidative stress (Panchuk et al., 2002).

**FIGURE 2:**
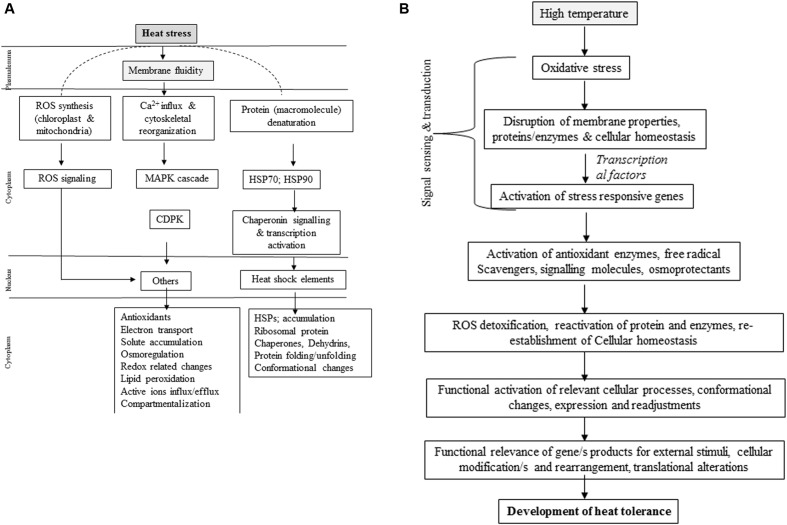
**(A)** Illustration of heat-stress tolerance mechanisms in plants. MAPK, Mitogen activated protein kinases; ROS, reactive oxygen species; HAMK, heat shock activate kinases; HSE, heat shock element; HSPs, heat shock proteins; CAPK, calcium dependent protein kinase; HSK, histidine kinase (Partly adapted form Wahid et al., 2007). **(B)** Schematic representation of heat induced signal trans duct ion and development of heat tolerance in plants (Partly adapted from Hasanuzzaman et al., 2013a).

Molecular approaches are being adopted for developing HT tolerance. At a cell level, heat stress causes changes in expression of genes involved in direct plant defense (Chinnusamy et al., 2007; Shinozaki and Yamaguchi-Shinozaki, 2007; Hasanuzzaman et al., 2012; Mathur et al., 2014) thereby regulating expression of osmoprotectants, detoxifying enzymes and regulatory proteins (Semenov and Halford, 2009). Heat stress is sensed by plants through increase in membrane fluidity, and the sensors located in membranes detect this phase transition resulting in conformational changes and phosphorylation/dephosphorylation events (Plieth et al., 1999). Membranes perceive heat stress activates calcium channels to release Ca^2+^ into the cystosol, combined with calmodulin activates calcium dependent kinases and various transcription factors (von Koskull-Doring et al., 2007; Zhang et al., 2009) (**Figure 2A**). Heat stress causes activation of unfolded proteins (UPR) of endoplasmic reticulum (ER-UPR) and cytosol (Cyt-UPR) (Mittler et al., 2012). Activated ER-UPR joins bZIP transcription factor that enters into nucleus and leads to expression of genes related to brassinosteriod signaling (Deng et al., 2013). Conversely, Cyt-UPR becomes activated by HSF capable of binding HSF-binding element at the promoter region of heat shock responsive genes (Sugio et al., 2009). HTs influence histone occupancy by changing H_2_A with H_2_A.Z in the nucleaosome in *Arabidopsis* (Kumar and Wigge, 2010), where actin-related protein-6 (ARP6) is engaged in replacing histone (Erkina et al., 2010). Heat stress increases membrane fluidity to induce lipid signaling to activate two molecules, phosphatidic acid (PA) and D-myo-inostiol-1,4,5-triphosphate (IP_3_) (Mishkind et al., 2009). IP_3_ and PA activate calcium channels located on ER to further release Ca^2+^ ions (Saidi et al., 2011). Based on duration and intensity of heat stress, release of Ca^2+^ ions and its expression pattern varies (calcium signatures). Calcium availability in cytosol during early hours of heat stress significantly reduced activation level of small HSP promoter and negatively influenced acquired thermo-tolerance. Heat stress does not trigger osmotic-responsive promoters, thus indicating heat- and osmo-sensors are specific, though they both require an influx of extracellular calcium. Such high specificity to heat and osmotic stresses possibly relies on specific effectors of calcium like calmodulin. Since heat stress in often associated with osmotic stress, high possibility of cross-talk between various secondary messengers of two stresses is expected while retaining their specificity (Saidi et al., 2009). Heat sensing and signaling need to be explored to test specific receptors in cell membrane, activation of HSFs, CaM kinases and occupancy of nucleosome. Knowing how phytohormones, especially having thermo-protective roles mediate and/or regulated through heat sensing would be useful in addressing heat tolerance from a practical view point (**Figure 2B**).

#### Transgenics for Heat Tolerance

Heat stress leads to production of special group of proteins termed as HSP’s, which on their molecular masses are further grouped into five classes; Hsp100, Hsp90, Hsp70, Hsp60 and small heat-shock proteins (sHsps). HSPs act as chaperones and prevent mis-folding and aggregation of native proteins. E.g., HSP101 from *Arabidopsis*, if over-expressed in rice plants, growth performance of rice was significantly enhanced during recovery from heat stress (Liu et al., 2011). The transcription of HSPs genes depends upon heat shock transcription factors, HSFs, which are located in the cytoplasm in inactive state and exist in three forms, HsfA1, HsfA2, HsfA3 (Tripp et al., 2009), which are vital to acquire thermo-tolerance as evident in *Arabidopsis* (Charng et al., 2007).

Quantitative trait loci mapping has established a genetic relationship with tolerance of various types of abiotic stresses and molecular markers helped in exploring QTLs linked to stress protection (Foolad, 2005). In *Arabidopsis*, four genomic loci associated with thermo-tolerance were reported while in maize, 11 QTLs related to pollen tube and germination under heat stress were mapped. A protein synthesis elongation factor Tu (EF-Tu) has been implicated in thermo-tolerance (Fu et al., 2012). A single plastid EF-Tu gene (tufA) is identified (Fu et al., 2012) in *Arabidopsis* and *Oryza sativa*, whereas multiple copies in nuclear genome of other plants, ranging from two genes (tufA and tufB) in *Nicotiana sylvestris* to four (tufA1, tufA2, tufB1, and tufB2) in *Glycine max* (Fu et al., 2012), indicating EF-Tu acts as chaperone and protects chloroplast stromal proteins from heat stress (Momcilovic and Ristic, 2004). Largely, over-expression of stress proteins resulted in enhanced thermo-tolerance in *Arabidopsis* (Alia et al., 1998), tobacco (Yang et al., 2005) and alfalfa (Saurez et al., 2008). Introduction of the *APX1* (ascorbate peroxidase) gene from pea and the *HvAPX1* gene from barley (Shi et al., 2001) in *Arabidopsis* led to improved thermo-tolerance.

### High Temperature Stress and Mungbean

From observations of the International Mungbean Nurseries, Poehlman (1978) suggested that a mean temperature of 28–30°C is optimum for this crop. Mungbean develops through nine distinct phenological phases as suggested by Carberry (2007) and Chauhan et al. (2010). The development of mungbean plants during stages 1–9 is mainly related to growing temperatures. The temperature requirement for different development stages is known as “thermal time” or day-degrees (unit °Cd) (Chauhan et al., 2010). The APSIM (Agricultural Production Systems sIMulator) mungbean model uses thermal time to drive phenological development and canopy expansion. The key temperatures used in thermal time calculations in the APSIM model were 7.5°C base, 30°C optimum and 40°C maximum (Carberry, 2007; Chauhan et al., 2010).

Under heat stress, activity of native housekeeping proteins in mungbean is suppressed while some specific proteins, known as heat shock proteins (Hsp70), are synthesized in leaves and in growing flower primordia (Schlesinger et al., 1982). HT (>40°C) has a direct effect on flower maintenance and pod formation (Kumari and Varma, 1983). Being a short-duration crop, possibly can be cultivated over a range of environments. Sensitivity of mungbean to varying photoperiod and temperature regimes necessitates developing thermo-photoperiod-insensitive varieties suit for both dry and wet seasons (Tickoo et al., 1996).

Among different stages, reproductive stage is most sensitive to HTs, resulting in loss of flower buds, pods and seed yield. The reproductive stage includes functioning of flowers to achieve pod set (viz., loss of pollen viability, germination, poor anther dehiscence, less pollen load on stigma and its sterility). The stigmatic surface also loses receptivity coupled with poor ovule viability (Kaushal et al., 2013). Therefore, pollen heat stress appears to be a crucial target for developing heat-tolerant mungbean (Rainey and Griffiths, 2005). Flower shedding is very common and the extent of flower shedding has been reported up to 79% (Kumari and Varma, 1983). However, little is known about HT driven floral and pod development mechanisms in mungbean (Tickoo et al., 1996). Khattak et al. (2009) reported no or less resistance to flower shedding under HTs. However, this relationship may not hold true for all genotypes (Aggarwal and Poehlman, 1977). Rawson and Craven (1979) reported temperature-flowering interactions in particular groups of genotypes with high mean temperatures (24–28°C) and long photoperiods (15–16 h). Similarly, 77 mutants derived from NM 92 and 51 recombinants selected from three crosses viz., VC1560D × NM92, VC1482C × NM92, and NM98 × VC3902A were also evaluated for this trait under HTs. No genotype showed absolute tolerance to flower shedding, while NM 92 showed susceptibility to the same trait under HTs (>40°C). Only the opened flowers shed under HTs and pods at any stage didn’t and humidity fluctuations had no effect on flower shedding^6^.

Several reasons for failure in reproductive growth and seed yield are attributed to heat stress. HT results in leaf scorching, leading to marginal (mild) to complete (severe) browning of leaves. The plant loses leaf pigments and photosynthetic function declines considerably (Kaushal et al., 2013; Awasthi et al., 2014). Leaf damage intensifies due to oxidative damage and reduction in antioxidative defense (Kumar et al., 2013). Truncated growth appears to be due to poor leaf expansion and growth. As reproductive parts (flowers, pods and seeds) rely on the leaves for sucrose and other macromolecules for nodulation and organelle function, maintenance of photosynthetic machinery of the leaves become vital under heat stress to sustain synthesis and transport of sucrose to these organs (Awasthi et al., 2014). Sucrose decreases in leaves and seeds owing to heat stress, which may link to reduced RuBisCO activity (or increased photorespiration) and sucrose synthesizing enzymes. Although useful information on the effect of heat stress on photosynthesis and photosynthesis exists for other crop plants, sparse in mungbean (Bindumadhava et al., 2015). Under HTs, RuBisCO though enzymatically active, RuBisCO activase (RA) suffers catalytic deactivation, which might trigger disruption of total turnover rates of the enzyme (Ray et al., 2003). Oxidative damage to leaves increases, impairing photosynthetic efficiency, which also affects nitrogen-fixing ability of mungbean rhizobia by restricting formation and spread of root hair (Bansal et al., 2014).

Terminal heat stress is a severe problem of mungbean in India, particularly in spring/summer. However, in the *kharif* crop, temperatures >40°C occur during early growth stage, causing a drastic reduction in seed yield due to pollen sterility, lack of fertilization and high or otherwise, complete flower shedding. Rainey and Griffiths (2005) reported abscission of reproductive organs as the primary determinant of yield under heat stress in many annual grain legumes. In subtropics or at higher altitudes, mungbean is sometime planted when mean night temperatures are <20°C – for instance, the spring/summer crop of northern India. In such environments, germination is delayed and reduced, plant growth becomes very slow. Hence, selection for rapid germination and growth would improve plant stand, promote early maturity and yield. Kumar et al. (2011) grew mungbean hydroponically at varying temperatures of 30/20°C (control), 35/25, 40/30, and 45/35°C (day/night; 12 h/12 h) with (50 μM) or without exogenous ASC to investigate effects on growth, membrane damage, leaf water status, components of oxidative stress and antioxidants. Among all the antioxidants, endogenous ASC content decreased maximum in 45/35°C grown plants, indicating its vital role in affecting the response of mungbean to heat stress. Exogenously applied ASC improves its endogenous levels along with glutathione and proline at 45/35°C. Thus indicating application of ASC overcomes heat stress-induced inhibition in growth and chlorosis triggered by oxidative stress.

To improve productivity of mungbean in warmer climates, it is crucial to decipher genetic variation for heat tolerance in the core germplasm and probe mechanisms governing therein. Recently, Kaur R. et al., 2015 elucidated the response of mungbean genotypes to heat stress on reproductive biology, leaf function and yield traits. Two genotypes (SML 832 and 668) were subjected to HTs (>40/25°C; day/night) during reproductive stage. A drastic reduction in pod set, number of filled pods (32–38%), seed number (43–47%) and seed yield (35–47%) was observed with no or less effect on phenology, flowering duration and podding. SML 668 was found to be more sensitive to heat stress than SML 832. This is perhaps the first preliminary report on the mechanisms affecting reproductive failures as a result of heat stress attributed to impaired sucrose metabolism in leaves and anthers. Malaviarachchi et al. (2016) assessed sensitivity of mungbean yield to increasing temperatures across different growing locations representing natural temperature gradient over two varied cropping seasons. In second season, crop experienced a broader temperature range, led to significantly reduced seed yield. However, insight on whether HT triggers failure in pollination, flower shedding, are they related to pollen infertility?, are responses linked to pollen viability and germination?, whether the ovule or the pollen more sensitive to heat?, if so to what extent, etc., are to be answered to know more on effects of heat stress on reproductive functions, pod maturity and final yield traits. Efforts must be made toward examining functional viability of pollen developed under heat stress for crossing ability with female parents grown under normal temperatures for both normal and heat stress conditions. We consider this is perhaps a current research gap and our own research is underway to address them in different growing environments.

Evidently, no studies on molecular mechanisms related to heat tolerance have been indicated, hence appropriate depiction of gene/s function and action against heat stress is still illusive. Apparently, apart from exploiting genetic variation for heat stress in various legumes, functional relevance of genes/gene product imparting tolerance are to be elucidated at molecular level. The existing high yielding genotypes of various legumes can be screened for heat tolerance either by planting them at hot spots or under late-sown conditions and selecting progenies on the bases of growth and yield traits. Diverse source viz., wild relatives might provide vital clues on target gene/s for heat tolerance which may have competitive advantage in breeding. Conclusively, integrated approaches involving marker-assisted selection, high throughput phenotyping and genotyping would form crucial links to unravel mechanisms of heat tolerance, which may subsequently pave the way for developing heat tolerant types in legumes through novel breeding approaches.

### Management Practices for Alleviating Heat Stress under Field Conditions

Although it is difficult to mitigate crop growth temperatures in open field, a few options can be applied with some success. There is a general crop management practice to adjust the method and date of sowing to ensure crops don’t face adverse heat effects during critical growth periods. However, in the literature, management practices for alleviating drought and heat stress are always presented together. We have attempted to de-link this association. The following soil management and irrigation practices, methods of handling crop residues and mulching, and choice of crops/varieties can alleviate negative effects of drought and heat stresses.

#### Soil Management and Irrigation

Changes in the soil surface affect soil water and heat balance in terms of soil water evaporation, infiltration and heat exchange between soil and atmosphere (Ferrero et al., 2005; Sekhon et al., 2010). These changes can be induced by tillage, surface residue management/mulching. The soil surface roughness, gradients in temperature and water vapor, and infiltration affect amount of water stored in soil and water uptake by plants (Lipiec et al., 2006, 2012). An increase in rooting depth in soils with definite hard subsoils can be attained by deep tillage. However, due to its high cost, practiced only in most dense soil paddocks (Siczek and Lipiec, 2011; Martínez et al., 2012). Use of surface organic mulch diminishes soil temperature for its low thermal conductivity (Khan et al., 2000) and favorably influence water content by controlling surface evaporation (Mulumba and Lal, 2008).

Modern irrigation techniques including sprinkling, drip and film hole irrigation save water (up to >50%) and improve grain yield and WUE compared to surface irrigation, but are less effective in terms of cost and energy requirements (Jensen, 2013).

#### Crop Management: Choice of Crops/Varieties and Sowing Date

Crops vary in their ability to tolerate drought and heat stress under water limited and HT conditions. Intrinsic genetic factors help plant to control these two stresses (Blum, 2005; Singh et al., 2010). Some crops/genotypes tolerate stress better than others do. In general, plant types and varieties that mature earlier perform better in drought-prone areas by escaping terminal drought (Singh et al., 2010). Moreover, crops and varieties with an ability to develop early crop stand and canopy structures perform better in drought- and heat-prone areas through reduction in soil evaporation and heating (Sekhon et al., 2010).

Waraich et al. (2012) highlighted that better plant nutrition can effectively alleviate adverse effects of temperature stress through a number of mechanisms. The management and regulated use of plant nutrients is very helpful to develop plant tolerance to temperature stress. HT stress results in increased generation of the ROS due to energy accumulation in stressed plants, which increases photo-oxidative effect and damage to chloroplast membranes. Addition of plant nutrients reduce toxicity of ROS by increasing concentration of antioxidants in plant cells. These antioxidants scavenge the ROS, maintain integrity of chloroplast membranes, and increase photosynthesis. These nutrients help to maintain high tissue water potential under temperature stress conditions^7^.

In temperate or subtropical zones, alteration in sowing date would help in increasing the probability that annual crop species will escape stressful HTs during sensitive stages of development. In some subtropical zones, the weather can be chilling in early spring and become progressively warmer, reaching very hot conditions in the middle of the summer (Ismail et al., 1997, 1999)^4^. Agroforestry, including concurrent production of trees and agricultural crops on the same piece of land, can be useful for sustaining stresses in the cropping zone (Khan et al., 2000). Change in temperatures and water limitations expected under climate change may have a significant effect on geographical distribution and occurrence of pests and diseases, as well as expansion of new pathogens limiting crop production (Vadez et al., 2011; Sharma, 2014), which implies a need for developing new control measures.

#### Exogenous Application of Thermo-Protectants

Recently, exogenous applications of protectants in the form of osmoprotectants (proline, glycine betaine, brassiosteroids, SA, phytohormones (ABA), signaling molecules, trace elements (selenium, etc.) and nutrients (phosphorus, potassium etc.) have been projected and found effective (Akhtar et al., 2015) in mitigating HT stress-induced damage in plants (Hasanuzzaman et al., 2010; Waraich et al., 2012)^3^. These molecules show promise in protecting plants from adverse effects of temperature stresses (Awasthi et al., 2015) and impart defense by managing the ROS through upregulation of antioxidant capacity.

In a few controlled experiments, application of proline and ASC conferred protection to heat-stressed plants of chickpea (Kaushal et al., 2011) and mungbean (Kumar et al., 2011), respectively. The positive effects of these molecules may be associated with reduction in damage to leaf and root tissues due to oxidative stress and activation of various antioxidants. Proline and ASC improved the growth of heat-stressed plants significantly, thus indicating the potential of these thermo-protectants. Wahid and Shabbir (2005) reported that, bean seeds pre-treated with glycine-betaine led to plants with lower membrane damage, better photosynthetic rate, improved leaf water potential and greater shoot dry mass compared to untreated seeds. In tomato, exogenous application of 4 mM spermidine improved heat resistance through better chlorophyll fluorescence properties, under heat stress^7^ and higher level of Ca^2+^ is required to mitigate adverse effects of the stress (Kleinhenz and Palta, 2002).

## Collaborative Research Of World Vegetable Center And Partners On Abiotic Stress In India

Development of mungbean genotypes tolerant to salinity and HT has been a long-standing goal of most institutes focusing on legume research. The World Vegetable Center’s mandate for mungbean research emphasizes establishing growth and yield records for elite accessions against salinity and HTs.

In Punjab regions of India and Pakistan, mungbean is grown during summer and *kharif* seasons. With introduction of short-duration varieties, production potential expanded to > 2 m ha. During spring, mungbean grown on about 60-80,000 hectares of area after potato, wheat, etc., is exposed to HTs, especially during reproductive stage. HT is detrimental to both vegetative and reproductive growth. Early maturing and short duration genotypes grown between rice-wheat cycles may experience temperatures >40°C, causing serious damage to intrinsic growth and yield performances.

To address them, we initiated a program for screening elite (45) lines [mungbean yellow mosaic disease (MYMD)-resistant] for salinity and HT under field and controlled conditions at ICRISAT Hyderabad, Panjab University (PU), Chandigarh, and Punjab Agricultural University (PAU), Ludhiana, India. The lines were from AVRDC’s own collection and from several Indian agricultural research institutes. Initial efforts yielded in identifying a few putative salinity (11 lines) and HT tolerant accessions (10 lines). The study continues to evaluate and validate these accessions under field conditions in different Indian states. These accessions will serve as donor parents in future mungbean breeding programs. Improving salinity along with heat tolerance would increase yield stability against harsh environments and possibly helps in expanding the geographical cultivable area.

## Inferences And Future Projections

Tolerance to salinity in legumes involves a multifaceted responses at cellular, molecular, physiological and whole-plant levels. Its adverse effects include osmotic stress, ion toxicity and nutrient imbalances. Although mungbean has intrinsic tolerance through physiological mechanisms, much needs to be explored in this species. Mungbean has distinct advantage of being a short-duration crop; it can grow in a range of soils and environments as a solo or as a relay crop. However, because it is sensitive to thermo-photoperiods and salinity, it has not been widely adopted by farmers. It is hoped that increasing osmotic stress tolerance would provide impetus for mungbean production under saline conditions.

Under HTs, modification of physiological and biochemical processes would gradually lead to heat tolerance through acclimation or adaptation. Depending on extremity and duration of intrinsic variations in plant types and allied environmental factors, mungbean would expect to show dynamic responses to stresses. However, most current experiments are laboratory and short duration investigations. Field experiments that explore different biochemical and molecular approaches as well as agronomic interventions are very much needed to gain tangibly HT responses on maturity and final yield patterns. At the field level, manipulating cultural practices can mitigate adverse effects of HT stress. In recent decades, exogenous applications of protectants and growth-promoting microorganisms have proven to be beneficial under HTs for their growth-promoting and antioxidant actions. Molecular approaches that reveal response/tolerance mechanisms will facilitate modifying plants that are able to withstand HTs without compromising yield. Integration of genes from closely related species for resistance to temperature and soil-related stresses should be the top priority for mungbean breeders to identify donor sources. Invariably, these qualities will substantiate the scope for horizontal expansion of mungbean and be a bonus in agricultural lands that remain fallow for 2–3 months after the harvest of the main (wheat/rice) crop.

Apart from exploring physiological and biochemical regulations of salinity and HT stress, there must be a continuous effort to compile a whole profile of genes, proteins, and metabolites responsible for different mechanisms of salinity and HT tolerance. Marker-linked genes for MYMD, powdery mildew, bruchid resistance, and some important yield traits already have been identified. However, potential sources of resistance to adverse climatic and soil conditions from wild relatives can also be integrated for crop improvement. With the availability of draft mungbean genome plus transcriptomic and metabolomics approaches, researchers are now well equipped to explore and exploit underlying molecular processes, which may pave the way to develop multi-stress tolerant mungbeans best suited to adverse growing environments.

## Author Contributions

BH made initial review framework and gather all information connected to abiotic stresses and implications. Refocused on effect of salinity and heat stress in legumes in general and mungbean in particular. Has made full efforts in writing, editing and revising the review. RN conceptualized the idea and supported in providing scientific literature linked to salinity and high temperatures from plant breeding point of view. He reviewed and revised the manuscript and helped to improve the reading and final editing. HN supported to gather recent research advancement in heat temperature tolerance in legumes, specially in mungbean as his team has been involved in this area from the last decade. He reviewed the manuscript and added valuable comments to improve it that helped in final editing.

## Conflict of Interest Statement

The authors declare that the research was conducted in the absence of any commercial or financial relationships that could be construed as a potential conflict of interest.

## Footnotes

1 www.knowledgebank.irri.org

2 http://plants.usda.gov

3 www.ncbi.nlm.nih.gov

4 www.plantstress.com

5 www.mdpi.com

6 www.pakbs.org

7 www.scielo.cl

## Abbreviations

ABA: abscisic acid
ASC: ascorbic acid
HT: high temperature
MDA: melondealdehyde
NaCl: sodium chloride
ROS: reactive oxygen species
RuBisCO: Rubilose 1,5 Bisphosphate Carboxylase/Oxygenase.
